# Boyd-Wong type contractions in generalized parametric bipolar metric space

**DOI:** 10.1016/j.heliyon.2024.e23998

**Published:** 2024-01-10

**Authors:** Manoj Kumar, Ozgur Ege, Vinit Mor, Pankaj Kumar, Manuel De la Sen

**Affiliations:** a Department of Mathematics, Baba Mastnath University, Asthal Bohar, Rohtak, 124021, Haryana, India; b Department of Mathematics, Faculty of Science, Ege University, Bornova, Izmir, 35100, Turkey; c Institute of Research and Development of Processes, Department of Electricity and Electronics, University of the Basque Country, 48940 Leioa, Spain

**Keywords:** primary, 47H10, secondary, 54H25, Boyd-Wong type contractions, Covariant mappings, Contravariant mappings, Fixed point, Gpbms (generalized parametric bipolar metric space)

## Abstract

In this manuscript, we introduce a new notion of generalized parametric bipolar metric space as a generalization of generalized parametric space and bipolar metric space. We also introduce Boyd-Wong type contractions for covariant and contravariant mappings to prove the fixed point results in the newly defined space. Some examples are also provided to illustrate the main results. Some corollaries are given of our results which shows the existence of fixed point for Banach type covariant and contravariant contractions. We solve integral and fractional differential equations with the help of proved results.

## 1 Introduction

Banach [20] developed the Banach Contraction Principle in 1922, which ensures the authenticity and uniqueness of a fixed point for contractions. In fact, prior to Banach, notable mathematicians such as Picard and Liouville utilized the fixed point approach to solve differential equations, in particular initial value problems. Several researchers have praised and researched the metric fixed point theory. These researchers are exploring this hypothesis for a variety of reasons and objectives. The natural and profound connection between theoretical results in nonlinear functional analysis and practical applications is the most fundamental reason why researchers feel it desirable to work on and examine metric fixed point theory.

With this motivation, several generalization of Banach contraction principle see ([7], [8], [9], [22]) as well as metric space ([21], [23], [26]) are carried out. Agarwal et al. proved many fixed point results in various spaces and use these results to solve boundary value problems and homotopy theory see ([2], [11], [24]). We shall mention only few of them which are helpful in designing the skeleton of our paper.

The following symbols are used throughout the paper:

(1) G,H - non empty sets,
(2) ω,B,P - distance functions,
(3) z - element of G,
(4) w - element of H,
(5) R,F - self maps on G∪H.

In 2016, a new type of metric function and space on two distinct sets was described by Mutlu and Gürdal [3] as follows:

Definition 1.1 [3] A bipolar metric is a function $\omega :G\times H\to R^{+}$ such that

(1) ω(z,w)=0 iff z=w for all (z,w)∈G×H;
(2) ω(z,w)=ω(w,z) for all z,w∈G∩H;
(3) $\omega (z_{1},w_{2})\leq \omega (z_{1},w_{1})+\omega (z_{2},w_{1})+\omega (z_{2},w_{2})$,

for all $z_{1},z_{2}\in G$ and $w_{1},w_{2}\in H$, where G,H are non-void sets. Then ordered triplet (G,H,ω) is termed bipolar metric space.

Definition 1.2 [3] Let $\left(G_{1},H_{1},\omega_{1}\right)$ and $\left(G_{2},H_{2},\omega_{2}\right)$ be two bipolar metric spaces and $R:G_{1}\cup H_{1}\to G_{2}\cup H_{2}$ be a function:

(1) R is called covariant mapping if $RG_{1}\subseteq G_{2}$ and $RH_{1}\subseteq H_{2}$ and is symbolized as $R:(G_{1},H_{1},\omega_{1})⇉(G_{2},H_{2},\omega_{2})$.
(2) R is called contravariant mapping if $RG_{1}\subseteq H_{2}$ and $RH_{1}\subseteq G_{2}$ and is symbolized $R:(G_{1},H_{1},\omega_{1})⇄(G_{2},H_{2},\omega_{2})$.

Since then, the authors have proved many fixed point theorems for covariant and contravariant mappings in bipolar metric spaces and applied these results to solve integral as well as fractional differential equations see ([4], [5], [6], [10], [16], [19], [25]).

In continuation of the generalization of notion of metric space, in 2014 Hussain et al. [14] using the non negative parameter *t* defined a new metric space known as parametric metric space and studied some fixed point results in the newly the defined space. After that many authors tried to generalize the parametric metric space using different approaches see ([12], [13][15], [17], [18]).

In 2016, Nihal Tas and Ozgur [15] introduced the notion of parametric *S*-metric space as a generalization of parametric space and proved some fixed point results for expansive mappings in it. In the same year later, Jain et al. [17] proved common and coupled fixed point theorems in parametric and parametric *b*-metric space.

In 2022, Usman et al. [12] generalized the parametric space by defining an equivalence relation on the set and called it *E*-parametric space.

In this respect, in 2023, Das and Bag gave the terminology of generalized parametric metric space as a generalization of parametric metric space by introducing the concept of binary operation at the place non-negative parameter *t* as mentioned below:

Definition 1.3 [1] Let a binary function $o:R^{+}\times R^{+}\to R^{+}$ such that for all $,\alpha ,\beta ,\gamma \in R^{+}$

(1) αo0=α;
(2) α≤β⇒αoγ≤βoγ;
(3) αoγ=γoα;
(4) αo(βoγ)=(αoβ)oγ.

Following are the examples of such binary operations:

(1) jok=max⁡{j,k};
(2) jok=j+k;
(3) $j\;o\;k={\left(j^{n}+k^{n}\right)}^{\frac{1}{n}}$, ∀n∈N.

Definition 1.4 [1] A generalized parametric metric is a function $P:G\times G\times (0,\infty )\to R^{+}$ such that

(1) (P(j,k,ϖ)=0, for all ϖ>0) iff j=k;
(2) P(j,k,ϖ)=P(k,j,ϖ) for all ϖ>0 and j,k∈G;
(3) For s,ϖ>0 and j,k,z∈G, P(j,k,s+ϖ)≤P(j,z,s)oP(k,z,ϖ),

where G is a non void set. Then ordered triplet (G,P,o) is called generalized parametric metric space.

Let Σ be a family of all functions W such that W:[0,∞)→[0,∞) is an upper semi-continuous from the right and also satisfies W(ϖ)<ϖ for ϖ>0.

The manuscript is divided into four sections. In section 1, we shall provide some basic notions from literature to build the next section which is section 2. In section 2, new notion of gpbms (generalized parametric bipolar metric space) is given and fixed point theorems for Boyd-Wong type contractions. In section 3, some consequences of our main results are discussed. In section 4, we solve an integral equation as well as fractional differential equation with the help of the theorems proved in section 2.

## 2 Results

In this section, we shall define generalized parametric bipolar metric space by generalizing the concept of bipolar given by Mutlu and Gürdal [3] in 2016 as well as generalized parametric space given by Das and Bag [1] in 2023. We also proved some fixed point results in the above mentioned space.

Definition 2.1 Let G, H(≠ϕ) and B:G×H×(0,∞)→[0,∞) be a function such that

(1) B(z,w,ϖ)=0 (for all ϖ>0) ⇔ z=w;
(2) B(z,w,ϖ)=B(w,z,ϖ) for all ϖ>0 with z,w∈G∩H;
(3) $B(z_{1},w_{2},r+s+t)\leq B(z_{1},w_{1},r)$ o $B(z_{2},w_{1},s)$ o $B(z_{2},w_{2},t)$;
  for all $z_{1},z_{2}\in G$ and $w_{1},w_{2}\in H$ and r,s,t>0.
  Then B is called gpbm and (G,H,B,o) is called gpbms.

If G∩H≠ϕ then space is called joint and if not, then disjoint. G is termed as left pole and H is termed as right pole. The elements of G∩H, H and G are called central, right and left elements.

Example 2.1 Let G=[0,2], H=[1,3] and define B:G×H×(0,∞)→[0,∞) as $B(z,w,\varpi )=\frac{|z-w{|}^{p}}{\varpi }$ for all ϖ>0 and $p\in (0,\frac{1}{2})$.

Define $a\;o\;b={\left(a^{2}+b^{2}\right)}^{\frac{1}{2}}$.

First two conditions of Definition 2.1 are easy to verify.

To prove the third condition, we use the following algebraic expression:

$${\left(a+b+c\right)}^{r}\leq a^{r}+b^{r}+c^{r},$$

for all a,b,c∈[0,∞) and r∈[0,1).

As $0<p<\frac{1}{2}$ so 0<2p<1.

By triangle inequality and above defined algebraic expression, we know that

$$|z_{1}-w_{2}|\leq |z_{1}-w_{1}|+|z_{2}-w_{1}|+|z_{2}-w_{2}|,|z_{1}-w_{2}{|}^{2p}\leq |z_{1}-w_{1}{|}^{2p}+|z_{2}-w_{1}{|}^{2p}+|z_{2}-w_{2}{|}^{2p},\frac{|z_{1}-w_{2}{|}^{2p}}{{\left(r+s+t\right)}^{2}}\leq \frac{|z_{1}-w_{1}{|}^{2p}+|z_{2}-w_{1}{|}^{2p}+|z_{2}-w_{2}{|}^{2p}}{{\left(r+s+t\right)}^{2}}\leq \frac{|z_{1}-w_{1}{|}^{2p}}{r^{2}}+\frac{|z_{2}-w_{1}{|}^{2p}}{s^{2}}+\frac{|z_{2}-w_{2}{|}^{2p}}{t^{2}},$$

for all r,s,t>0.

So, (G,H,B,o) is a gpbms.

Example 2.2 Let $G=U_{m}(R)$, $H=L_{m}(R)$ and define B:G×H×(0,∞)→[0,∞) as $B(G,H,\varpi )=\sum_{i,j=1}^{m}|z_{ij}-w_{ij}{|}^{p}$ for all ϖ>0 and $p\in (0,\frac{1}{2})$.

Define aob=a+b.

First two conditions are easy to check. By using the following algebraic identity the proof of 3rd condition follows the same steps like Example 2.1.

$${\left(a+b\right)}^{r}\leq a^{r}+b^{r},$$

for all a,b,c∈[0,∞) and r∈[0,1).

So, (G,H,B,o) is a gpbms.

Definition 2.2 Let (G,H,B,o) be a gpbms.

(1) Any sequence $(z_{n})\subseteq G$ is known as left sequence and we say that it is convergent to w∈H when for all ϖ>0, $B(z_{n},w,\varpi )\to 0$ as n→∞ and for a right sequence $(w_{n})\subseteq H$ we say that it is convergent to z∈G when for all ϖ>0 $B(z,w_{n},\varpi )\to 0$ as n→∞.
(2) The ordered pair $\left\{(z_{n},w_{n})\right\}$ in G×H is termed as bisequence on (G,H,B,o).
(3) If for the bisequence $\left\{(z_{n},w_{n})\right\}$ sequences $\left(z_{n}\right)$ and $\left(w_{n}\right)$ converge, then bisequence is known as convergent. If both the sequences $\left(z_{n}\right)$ and $\left(w_{n}\right)$ have the common limit point then the bisequence $\left(z_{n},w_{n}\right)$ is known as biconvergent.
(4) A bisequence $\left\{(z_{n},w_{n})\right\}$ on (G,H,B,o) is termed as Cauchy bisequence, if there exists a positive integer N∈N such that $B(z_{n},w_{m},\varpi )<\epsilon$ for all n,m≥N, ϵ>0 and ϖ>0.
(5) If every Cauchy bisequence is convergent then gpbms is said to be complete.

Definition 2.3 Let $\left(G_{1},H_{1},B_{1},o\right)$ and $\left(G_{2},H_{2},B_{2},o\right)$ be two gpbms.

(1) The left continuity of a covariant map $F:(G_{1},H_{1},B_{1},o)⇉(G_{2},H_{2},B_{2},o)$ exits at a point $z_{0}\in G$ if for all ϵ,ϖ>0 we have δ>0 in such a way that $B_{2}(Fz_{0},Fw,\varpi )<\epsilon$ when $B_{1}(z_{0},w,\varpi )<\delta$.
(2) The right continuity of a covariant map $F:(G_{1},H_{1},B_{1},o)⇉(G_{2},H_{2},B_{2},o)$ at a point $w_{0}\in H$ if for all ϵ,ϖ>0 we have δ>0 in such a way that $B_{2}(Fz,Fw_{0},\varpi )<\epsilon$ when $B_{1}(z,w_{0},\varpi )<\delta$.
(3) The continuity of a covariant map F exists at $\left(z_{0},w_{0}\right)$ if left continuity exists at $z_{0}$ and right continuity at $w_{0}$.
(4) The continuity of a contravariant map $F:(G_{1},H_{1},B_{1},o)⇄(G_{2},H_{2},B_{2},o)$ exists iff continuity of F as a covariant map $F:(G_{1},H_{1},B_{1},o)⇉(G_{2},H_{2},B_{2},o)$ exists.

Proposition 2.1 *If the bisequence* $\left\{(z_{n},w_{n})\right\}$ *has a common limit* u∈G∩H *then each sequence* $\left(z_{n}),(w_{n}\right)$ *have a unique limit.*

Proof Suppose if possible $z_{n}\to v\in H$ as n→∞.

Then, $B(u,v,r+s+t)\leq B(u,u,r)\;o\;B(z_{n},u,r)\;o\;B(z_{n},v,r)$

So, limit n→∞ gives B(u,v,r+s+t)=0 for all r,s,t>0.

Hence, u=v. □

Now, in the next theorem we establish the existence and uniqueness of fixed point for the Boyd-Wong type covariant contractions.

Theorem 2.3 *Let*(G,H,B,o)*be a complete gpbms and*F:(G,H)⇉(G,H)*is a covariant map such that*

(1) B(Fz,Fw,ϖ)≤W(B(z,w,ϖ)),

*for all*ϖ>0*,*(z,w)∈G×H*and*W∈Σ*.*

*Then*F*has a unique fixed point.*

Proof Let $z_{0}\in G$ and $w_{0}\in H$. Construct two sequences $(z_{n})\in G$ and $(w_{n})\in H$ such that $z_{n+1}=Fz_{n}$, $w_{n+1}=Fw_{n}$, for all n∈N.

Then $\left\{(z_{n},w_{n})\right\}$ is a bisequence in G×H.

Now, for $z=z_{n-1}$, $w=w_{n-1}$ and ϖ>0, equation (1) shows that

$$a_{n}(\varpi )=B(z_{n},w_{n},\varpi )=B(Fz_{n-1},Fw_{n-1},\varpi )\leq W(B(z_{n-1},w_{n-1},\varpi )),<B(z_{n-1},w_{n-1},\varpi ),=a_{n-1}(\varpi ).$$

This implies that $\left\{a_{n}(\varpi )\right\}$ is a strictly non increasing sequence of $R^{+}$ and hence convergent. Suppose, $a_{n}(\varpi )\to a(\varpi )$ as n→∞. Now, we shall show that a(ϖ)=0 for all ϖ>0. On the contrary, suppose there is $\varpi_{0}>0$ with $a(\varpi_{0})>0$. Then $a_{n+1}(\varpi )\leq W(a_{n})(\varpi )$ and upper semi-continuity from right of W gives,

$$a(\varpi_{0})\leq \lim_{a_{n}(\varpi_{0})\to a{\left(\varpi_{0}\right)}^{+}}W(a_{n}(\varpi_{0}))\leq W(a(\varpi_{0})).$$

But this contradicts that for all ϖ>0, W(ϖ)<ϖ. Thus, we get

(2) $$\lim_{n\to \infty }a_{n}(\varpi )=\lim_{n\to \infty }B(F^{n}z_{0},F^{n}w_{0},\varpi )=0,$$

for all ϖ>0.

Now, for $z=z_{n-1}$, $w=w_{n}$ and ϖ>0, equation (1) implies that

$$b_{n}(\varpi )=B(z_{n},w_{n+1},\varpi )=B(Fz_{n-1},Fw_{n},\varpi )\leq W(B(z_{n-1},w_{n},\varpi )),<B(z_{n-1},w_{n},\varpi ),=b_{n-1}(\varpi ).$$

This implies that $\left\{b_{n}(\varpi )\right\}$ is a strictly non increasing sequence of $R^{+}$ and hence convergent. Suppose, $b_{n}(\varpi )\to b(\varpi )$ as n→∞. Now, we shall show that b(ϖ)=0 for all ϖ>0. On the contrary, if there is $\varpi_{0}>0$ with $b(\varpi_{0})>0$. Then $b_{n+1}(\varpi )\leq W(b_{n})(\varpi )$ and upper semi-continuity from right of W gives,

$$b(\varpi_{0})\leq \lim_{b_{n}(\varpi_{0})\to b{\left(\varpi_{0}\right)}^{+}}W(b_{n}(\varpi_{0}))\leq W(b(\varpi_{0})).$$

But this contradicts that for all ϖ>0, W(ϖ)<ϖ. Thus we get,

(3) $$\lim_{n\to \infty }b_{n}(\varpi )=\lim_{n\to \infty }B(F^{n-1}z_{0},F^{n}w_{0},\varpi )=0,$$

for all ϖ>0.

Now, we shall prove that $\left\{(z_{n},w_{n})\right\}$ is a Cauchy bisequence.

By using Definition of gpbms, equations (2) and (3), for all ϖ>0 and n,p∈N, we have

$$B(z_{n},w_{n+p},\varpi )\leq B(z_{n},w_{n+1},\frac{\varpi }{3})\;o\;B(z_{n+1},w_{n+1},\frac{\varpi }{3})\;o\;B(z_{n+1},w_{n+p},\frac{\varpi }{3}),\leq B(z_{n},w_{n+1},\frac{\varpi }{3})\;o\;B(z_{n+1},w_{n+1},\frac{\varpi }{3}),oB(z_{n+1},w_{n+2},\frac{\varpi }{9})\;o\;B(z_{n+2},w_{n+2},\frac{\varpi }{9})\;o\;B(z_{n+2},w_{n+p},\frac{\varpi }{9})...\leq B(z_{n},w_{n+1},\frac{\varpi }{3})\;o\;...\;o\;B(z_{n+p-1},w_{n+p},\frac{\varpi }{3^{p-1}}).$$

Applying limit as n→∞, the above inequality becomes

(4) $$\lim_{n\to \infty }B(z_{n},w_{n+p},\varpi )=0.$$

Again, by using Definition of gpbms, for all ϖ>0 and n,p∈N, we have

$$B(z_{n+p},w_{n},\varpi )\leq B(z_{n+p},w_{n+1},\frac{\varpi }{3})\;o\;B(z_{n},w_{n+1},\frac{\varpi }{3})\;o\;B(z_{n},w_{n},\frac{\varpi }{3}),\leq B(z_{n},w_{n+1},\frac{\varpi }{3})\;o\;B(z_{n},w_{n},\frac{\varpi }{3}),oB(z_{n+p},w_{n+2},\frac{\varpi }{9})\;o\;B(z_{n+1},w_{n+1},\frac{\varpi }{9})\;o\;B(z_{n+1},w_{n+2},\frac{\varpi }{9}),...\leq B(z_{n},w_{n},\frac{\varpi }{3})\;o\;...\;o\;B(z_{n+p},w_{n+p},\frac{\varpi }{3^{p}}).$$

Applying limit as n→∞, the above inequality becomes

(5) $$\lim_{n\to \infty }B(z_{n+p},w_{n},\varpi )=0.$$

From equations (4) and (5), we have $\left\{(z_{n},w_{n})\right\}$ is a Cauchy bisequence.

Since (G,H,B,o) is a complete gpbms, so by Proposition 2.1, $\left\{(z_{n},w_{n})\right\}$ biconverges to a point u∈G∩H that is $\lim_{n\to \infty }z_{n}=\lim_{n\to \infty }w_{n}=u$.

So, by continuity of F, we have $\mathrm{Fu}=\lim_{n\to \infty }Fz_{n}=\lim_{n\to \infty }z_{n+1}=u$.

Thus Fu=u.

Now, we establish that fixed point *u* for F is unique.

If possible, suppose that F have *u* and *v* two distinct fixed points.

Then

B(u,v,ϖ)=B(Fu,Fv,ϖ)≤W(B(u,v,ϖ))<B(u,v,ϖ),

for all ϖ>0.

This implies that u=v. □

In the next theorem we show the existence of unique fixed point for Boyd-Wong type contravariant contractions.

Theorem 2.4 *Let*(G,H,B,o)*be a complete gpbms and*F:(G,H)⇄(G,H)*is a covariant map such that*

(6) B(Fw,Fz,ϖ)≤W(B(z,w,ϖ)),

*for all*ϖ>0*and*(z,w)∈G×H*and*W∈Σ*.*

*Then,*F*has a unique fixed point.*

Proof Let $z_{0}\in G$ and $w_{0}\in H$. Construct two sequences $(z_{n})\in G$ and $(w_{n})\in H$ such that $w_{n}=Fz_{n}$, $z_{n+1}=Fw_{n}$, for all n∈N.

Then $\left\{(z_{n},w_{n})\right\}$ is a bisequence in G×H.

From equation (6) we have

(7) $$a_{n}(\varpi )=B(z_{n},w_{n},\varpi )=B(Fw_{n-1},Fz_{n},\varpi )\leq W(B(z_{n},w_{n-1},\varpi )),<B(z_{n},w_{n-1},\varpi ).$$

Again, equation (6) gives

(8) $$b_{n}(\varpi )=B(z_{n+1},w_{n},\varpi )=B(Fw_{n},Fz_{n},\varpi )\leq W(B(z_{n},w_{n},\varpi )),<B(z_{n},w_{n},\varpi ).$$

From equation (7) and (8), we can say that $\left\{a_{n}(\varpi )\right\}$ is a strictly non increasing sequence of $R^{+}$ and hence convergent. Suppose, $a_{n}(\varpi )\to a(\varpi )$ as n→∞. Now, we shall show that a(ϖ)=0 for all ϖ>0. On the contrary, suppose there is $\varpi_{0}>0$ with $a(\varpi_{0})>0$.

Upper semi-continuity from right of W gives

$$a(\varpi_{0})\leq \lim_{a_{n}(\varpi_{0})\to a{\left(\varpi_{0}\right)}^{+}}W(a_{n}(\varpi_{0}))\leq W(a(\varpi_{0}))<a(\varpi_{0}),$$

a contradiction.

Thus, we get

(9) $$\lim_{n\to \infty }a_{n}(\varpi )=0,$$

for all ϖ>0.

Similarly, $\left\{b_{n}(\varpi )\right\}$ is a strictly non increasing sequence of positive reals and hence convergent. Suppose, $b_{n}(\varpi )\to b(\varpi )$ as n→∞. Now, we shall show that b(ϖ)=0 for all ϖ>0. On the contrary, let us assume that there exists $\varpi_{0}>0$ such that $b(\varpi_{0})>0$. Upper semi-continuity from right of W gives

$$b(\varpi_{0})\leq \lim_{b_{n}(\varpi_{0})\to b{\left(\varpi_{0}\right)}^{+}}W(b_{n}(\varpi_{0}))\leq W(b(\varpi_{0}))<b(\varpi_{0}),$$

a contradiction. Thus, we get

(10) $$\lim_{n\to \infty }b_{n}(\varpi )=0,$$

for all ϖ>0.

Now, we shall prove that $\left\{(z_{n},w_{n})\right\}$ is a Cauchy bisequence.

By using Definition of gpbms, equations (9) and (10), for all ϖ>0 and n,p∈N, we have

$$B(z_{n+p},w_{n},\varpi )\leq B(z_{n+p},w_{n+1},\frac{\varpi }{3})\;o\;B(z_{n+1},w_{n+1},\frac{\varpi }{3})\;o\;B(z_{n+1},w_{n},\frac{\varpi }{3}),\leq B(z_{n+1},w_{n+1},\frac{\varpi }{3})\;o\;B(z_{n+1},w_{n},\frac{\varpi }{3}),oB(z_{n+p},w_{n+2},\frac{\varpi }{9})\;o\;B(z_{n+2},w_{n+2},\frac{\varpi }{9})\;o\;B(z_{n+2},w_{n+1},\frac{\varpi }{9}),...\leq B(z_{n+1},w_{n},\frac{\varpi }{3})\;o\;...\;o\;B(z_{n+p-1},w_{n+p-1},\frac{\varpi }{3^{p-1}}).$$

Taking limit as n→∞, the above inequality becomes

(11) $$\lim_{n\to \infty }B(z_{n+p},w_{n},\varpi )=0.$$

Again, by using Definition of gpbms, for all ϖ>0 and n,p∈N, we have

$$B(z_{n},w_{n+p},\varpi )\leq B(z_{n},w_{n},\frac{\varpi }{3})\;o\;B(z_{n+1},w_{n},\frac{\varpi }{3})\;o\;B(z_{n+1},w_{n+p},\frac{\varpi }{3}),\leq B(z_{n+1},w_{n},\frac{\varpi }{3})\;o\;B(z_{n},w_{n},\frac{\varpi }{3}),oB(z_{n+1},w_{n+1},\frac{\varpi }{9})\;o\;B(z_{n+2},w_{n+1},\frac{\varpi }{9})\;o\;B(z_{n+2},w_{n+p},\frac{\varpi }{9}),...\leq B(z_{n},w_{n},\frac{\varpi }{3})\;o\;...\;o\;B(z_{n+p},w_{n+p-1},\frac{\varpi }{3^{p-1}}).$$

Taking limit as n→∞, the above inequality becomes

(12) $$\lim_{n\to \infty }B(z_{n},w_{n+p},\varpi )=0.$$

From equations (11) and (12), $\left\{(z_{n},w_{n})\right\}$ is a Cauchy bisequence.

Since (G,H,B,o) is a complete gpbms, so by Proposition 2.1, $\left\{(z_{n},w_{n})\right\}$ biconverges to a point u∈G∩H that is $\lim_{n\to \infty }z_{n}=\lim_{n\to \infty }w_{n}=u$.

Now, by continuity of F, we have $\lim_{n\to \infty }z_{n}=u$ implies $\lim_{n\to \infty }Fz_{n}=\mathrm{Fu}=\lim_{n\to \infty }w_{n}=u$

So, Fu=u.

Now, we establish that fixed point *u* for F is unique.

If possible, assume that F have two distinct fixed points *u* and *v*. Then

B(u,v,ϖ)=B(Fu,Fv,ϖ)≤W(B(u,v,ϖ))<B(u,v,ϖ),

for all ϖ>0. This implies that u=v. □

In the above two proved results, Boyd-Wong type's covariant and contravariant maps are continuous. But in the next two theorems, we replace B(z,w,ϖ) by M(z,w,ϖ). So, now we need the continuity of the maps.

Theorem 2.5 *Let*(G,H,B,o)*be a complete gpbms and*F:(G,H)⇄(G,H)*be a continuous contravariant map such that*

(13) B(Fw,Fz,ϖ)≤W(M(z,w,ϖ)),

*where*M(z,w,ϖ)=max⁡{B(z,w,ϖ),B(z,Fz,ϖ),B(Fw,w,ϖ)}*for all*(z,w)∈G×H*and*W∈Σ*.*

*Then*F*possesses a unique fixed point.*

Proof Let $z_{0}\in G$. Then assume two iterated sequences $z_{n+1}=Fw_{n},Fz_{n}=w_{n}$ for all n∈N.

Then $\left\{(z_{n},w_{n})\right\}$ is a bisequence in G×H.

For $z=z_{n}$ and $w=w_{n-1}$ equation (13) becomes

(14) $$a_{n}(\varpi )=B(z_{n},w_{n},\varpi )=B(fw_{n-1},Fz_{n}.\varpi )\leq W(M(z_{n},w_{n-1},\varpi )),$$

where

$$M(z_{n},w_{n-1},\varpi )=\max\{B(z_{n},w_{n-1},\varpi ),B(z_{n},Fz_{n},\varpi ),B(Fw_{n-1},w_{n-1},\varpi )\},=\max\{B(z_{n},w_{n-1},\varpi ),B(z_{n},w_{n},\varpi ),B(z_{n},w_{n-1},\varpi )\}.$$

Suppose that $B(z_{n},w_{n},\varpi )\geq B(z_{n},w_{n-1},\varpi )$ then equation (14) implies that

$$B(z_{n},w_{n},\varpi )\leq W(B(z_{n},w_{n},\varpi ))<B(z_{n},w_{n},\varpi ),$$

a contradiction.

So,

(15) $$B(z_{n},w_{n},\varpi )\leq B(z_{n},w_{n-1},\varpi ),$$

for all ϖ>0.

Similarly for $z=z_{n},w=w_{n}$ equation (13) implies that

(16) $$b_{n}(\varpi )=B(z_{n+1},w_{n},\varpi )=B(Fw_{n},Fz_{n},\varpi )\leq W(M(z_{n},w_{n},\varpi )),$$

where

$$M(z_{n},w_{n},\varpi )=\max\{B(z_{n},w_{n},\varpi ),B(z_{n},Tz_{n},\varpi ),B(Tw_{n},w_{n},\varpi )\},=\max\{B(z_{n},w_{n},\varpi ),B(z_{n},w_{n},\varpi ),B(z_{n+1},w_{n},\varpi )\}.$$

Suppose, if possible that

$$B(z_{n+1},w_{n},\varpi )\geq B(z_{n},w_{n},\varpi ).$$

Then equation (16) implies that

$$B(z_{n+1},w_{n},\varpi )\leq W(B(z_{n+1},w_{n},\varpi ))<B(z_{n+1},w_{n},\varpi ),$$

a contradiction.

So,

(17) $$B(z_{n+1},w_{n},\varpi )\leq B(z_{n},w_{n},\varpi ),$$

for all ϖ>0.

From equation (15) and (17) we obtain that $\left\{a_{n}(\varpi )\right\}$ and $\left\{b_{n}(\varpi )\right\}$ are monotonically decreasing sequences of $R^{+}$ and hence convergent.

Claim $a_{n}(\varpi )\to a(\varpi )=0$*and*$b_{n}(\varpi )\to b(\varpi )=0$*as*n→∞*.*

On the contrary, suppose there is $\varpi_{0}>0$ such that $a(\varpi_{0})>0$.

Upper semi-continuity from right of W gives

$$a(\varpi_{0})\leq \lim_{a_{n}(\varpi_{0})\to a{\left(\varpi_{0}\right)}^{+}}W(a_{n}(\varpi_{0}))\leq W(a(\varpi_{0}))<a(\varpi_{0}),$$

a contradiction. Thus, we get

(18) $$\lim_{n\to \infty }a_{n}(\varpi )=0,$$

for all ϖ>0. Similarly, we can say that

(19) $$\lim_{n\to \infty }b_{n}(\varpi )=0,$$

for all ϖ>0.

Now, we prove that $\left\{(z_{n},w_{n})\right\}$ is a Cauchy bisequence. Suppose that $\left\{(z_{n},w_{n})\right\}$ is not Cauchy bisequence, for some ϵ>0 there are subsequences n(x) and m(x) of $Z^{+}$ such that n(x)>m(x)>x.

(20) $$B(z_{n(x)},w_{m(x)},\varpi )\geq \epsilon ,$$

for all ϖ>0. We take n(x) in such a way that it is the smallest integer satisfying equation (20), it means

(21) $$B(z_{n(x)-1},w_{m(x)},\varpi )\leq \epsilon ,$$

for all ϖ>0.

So,

$$\epsilon \leq B(z_{n(x)},w_{m(x)},\varpi )\leq B(z_{n(x)},w_{n(x)-1},\varpi )\;o\;B(z_{n(x)-1},w_{n(x)-1},\varpi )\;o\;B(z_{n(x)-1},w_{m(x)},\varpi ).$$

Taking limit x→∞ and using equations (18)-(19), (21) and ao0=a above becomes

(22) $$\lim_{x\to \infty }B(z_{n(x)},w_{m(x)},\varpi )=\epsilon .$$

By the Definition of gpbms

$$\epsilon \leq B(z_{n(x)},w_{m(x)},\varpi )\leq B(z_{n(x)},w_{n(x)},\frac{\varpi }{3}\;o\;B(z_{m(x)+1},w_{n(x)},\frac{\varpi }{3})\;o\;B(z_{m(x)},w_{m(x)},\frac{\varpi }{3}),$$

and

$$B(z_{m(x)+1},w_{n(x)},\frac{\varpi }{3})\leq B(z_{m(x)+1},w_{m(x)},\frac{\varpi }{9}\;o\;B(z_{n(x)},w_{m(x)},\frac{\varpi }{9})\;o\;B(z_{n(x)},w_{n(x)},\frac{\varpi }{9}),$$

taking x→∞, and using equations (18)-(19) and (22), we get

(23) $$\lim_{x\to \infty }B(z_{m(x)+1},w_{n(x)},\frac{\varpi }{3})=\epsilon ,$$

for all $\frac{\varpi }{3}>0\Rightarrow \varpi >0$.

Now,

(24) $$B(z_{m(x)+1},w_{n(x)},\varpi )=B(z_{n(x)+1},w_{m(x)},\varpi )\leq W(M(z_{n(x)},w_{m(x)},\varpi )),$$

where

$$M(z_{n(x)},w_{m(x)},\varpi )=\max\{B(z_{n(x)},w_{m(x)},\varpi ),B(z_{n(x)},Tz_{n(x)},\varpi ),B(Tw_{m(x)},w_{m(x)},\varpi )\},=\max\{B(z_{n(x)},w_{m(x)},\varpi ),B(z_{n(x)},w_{n(x)},\varpi ),B(z_{m(x)+1},w_{m(x)},\varpi )\}.$$

Using the above inequality in equation (24) and taking x→∞ with the help of equation (23), we get

ϵ≤W(max{ϵ,0,0})=ϕ(ϵ)<ϵ,

a contradiction.

So, $\left\{(z_{n},w_{n})\right\}$ is a Cauchy bisequence. Since the space is complete, hence by Proposition 2.1, $\left\{(z_{n},w_{n})\right\}$ is biconvergent that is there exists u∈G∩H such that $z_{n}\to u$ and $w_{n}\to u$ as n→∞.

Now, by continuity of F, we have $\lim_{n\to \infty }z_{n}=u$ implies

(25) $$\lim_{n\to \infty }Fz_{n}=\mathrm{Fu}=\lim_{n\to \infty }w_{n}=u.$$

So, equation (25) implies that Fu=u.

Uniqueness: If possible suppose that *u* and *v* are distinct fixed points.

(26) B(u,v,ϖ)=B(Fu,Fv,ϖ)≤W(M(u,v,ϖ)),

where

M(u,v,ϖ)=max⁡{B(u,v,ϖ),B(u,Fu,ϖ),B(Fu,v,ϖ)},=max⁡{B(u,v,ϖ),o,o},=B(u,v,ϖ).

Putting in equation (26), we get

B(u,v,ϖ)≤W(B(u,v,ϖ))<B(u,v,ϖ),

a contradiction.

This implies that u=v. □

In the next theorem, we weaken the hypothesis of Theorem 2.5 by introducing more factors in max term.

Theorem 2.6 *Let*(G,H,B,o)*be a complete gpbms and*F:(G,H)⇄(G,H)*be a continuous contravariant map such that*

(27) B(Fw,Fz,ϖ)≤W(M(z,w,ϖ)),

*where*

$$M(z,w,\varpi )=\max\{B(z,w,\varpi ),B(z,Fz,\varpi ),B(Fw,w,\varpi ),\frac{B(z,Fz,\varpi )+B(Fw,w,\varpi )}{2},\frac{B(z,Fz,\varpi )\cdot B(Fw,w,\varpi )}{B(z,w,\varpi )},\frac{B(z,Fz,\varpi )(1+B(Fw,w,\varpi ))}{1+B(z,w,\varpi )}\},$$

*for all*(z,w)∈G×H*and*W∈Σ*.*

*Then*F*has a unique fixed point.*

Proof Let $z_{0}\in G$. Then assume two iterated sequences $z_{n+1}=Fw_{n},Fz_{n}=w_{n}$ for all n∈N.

Then $\left\{(z_{n},w_{n})\right\}$ is a bisequence in G×H.

For $z=z_{n}$ and $w=w_{n-1}$ equation (27) becomes

(28) $$a_{n}(\varpi )=B(z_{n},w_{n},\varpi )=B(fw_{n-1},Fz_{n}.\varpi )\leq W(M(z_{n},w_{n-1},\varpi )),$$

where

$$M(z_{n},w_{n-1},\varpi )=\max\{B(z_{n},w_{n-1},\varpi ),B(z_{n},Fz_{n},\varpi ),B(Fw_{n-1},w_{n-1},\varpi ),\frac{B(z_{n},Fz_{n},\varpi )+B(Fw_{n-1},w_{n-1},\varpi )}{2},\frac{B(z_{n},Fz_{n},\varpi )\cdot B(Fw_{n-1},w_{n-1},\varpi )}{B(z_{n},w_{n-1},\varpi )},\frac{B(z_{n},Fz_{n},\varpi )\cdot (1+B(Fw_{n-1},w_{n-1},\varpi ))}{1+B(z_{n},w_{n-1},\varpi )}\},=\max\{B(z_{n},w_{n-1},\varpi ),B(z_{n},w_{n},\varpi ),B(z_{n},w_{n-1},\varpi ),\frac{B(z_{n},w_{n},\varpi )+B(z_{n},w_{n-1},\varpi )}{2},\frac{B(z_{n},w_{n},\varpi )\cdot B(z_{n},w_{n-1},\varpi )}{B(z_{n},w_{n-1},\varpi )},\frac{B(z_{n},w_{n},\varpi )\cdot (1+B(z_{n},w_{n-1},\varpi ))}{1+B(z_{n},w_{n-1},\varpi )}\},=\max\{B(z_{n},w_{n-1},\varpi ),B(z_{n},w_{n},\varpi )\}.$$

Suppose that $B(z_{n},w_{n},\varpi )\geq B(z_{n},w_{n-1},\varpi )$ then equation (28) implies that

$$B(z_{n},w_{n},\varpi )\leq W(B(z_{n},w_{n},\varpi ))<B(z_{n},w_{n},\varpi ),$$

a contradiction.

So,

(29) $$B(z_{n},w_{n},\varpi )\leq B(z_{n},w_{n-1},\varpi ),$$

for all ϖ>0.

Similarly for $z=z_{n},w=w_{n}$ equation (27) implies that

(30) $$b_{n}(\varpi )=B(z_{n+1},w_{n},\varpi )=B(Fw_{n},Fz_{n},\varpi )\leq W(M(z_{n},w_{n},\varpi )),$$

where

$$M(z_{n},w_{n},\varpi )=\max\{B(z_{n},w_{n},\varpi ),B(z_{n},Fz_{n},\varpi ),B(Fw_{n},w_{n},\varpi ),\frac{B(z_{n},Fz_{n},\varpi )+B(Fw_{n},w_{n},\varpi )}{2},\frac{B(z_{n},Fz_{n},\varpi )\cdot B(Fw_{n},w_{n},\varpi )}{B(z_{n},w_{n},\varpi )},\frac{B(z_{n},Fz_{n},\varpi )\cdot (1+B(Fw_{n},w_{n},\varpi ))}{1+B(z_{n},w_{n},\varpi )}\}=\max\{B(z_{n},w_{n},\varpi ),B(z_{n},w_{n},\varpi ),B(z_{n+1},w_{n},\varpi ),\frac{B(z_{n},w_{n},\varpi )+B(z_{n+1},w_{n},\varpi )}{2},\frac{B(z_{n},w_{n},\varpi )\cdot B(z_{n+1},w_{n},\varpi )}{B(z_{n},w_{n},\varpi )},\frac{B(z_{n},w_{n},\varpi )\cdot (1+B(z_{n+1},w_{n},\varpi ))}{1+B(z_{n},w_{n},\varpi )}\},=\max\{B(z_{n+1},w_{n},\varpi ),B(z_{n},w_{n},\varpi )\}.$$

Suppose if possible that

$$B(z_{n+1},w_{n},\varpi )\geq B(z_{n},w_{n},\varpi ).$$

Then equation (30) implies that

$$B(z_{n+1},w_{n},\varpi )\leq W(B(z_{n+1},w_{n},\varpi ))<B(z_{n+1},w_{n},\varpi ),$$

a contradiction.

So,

(31) $$B(z_{n+1},w_{n},\varpi )\leq B(z_{n},w_{n},\varpi ),$$

for all ϖ>0.

From equation (28) and (30) with the help of equations (29) and (31), we obtain that $\left\{a_{n}(\varpi )\right\}$ and $\left\{b_{n}(\varpi )\right\}$ are non increasing sequences of $R^{+}$ and hence convergent.

Claim $a_{n}(\varpi )\to a(\varpi )=0$ *and* $b_{n}(\varpi )\to b(\varpi )=0$ *as* n→∞ *On the contrary, suppose there is some* $\varpi_{0}>0$ *with* $a(\varpi_{0})>0$ *.*

Upper semi-continuity from right of W gives

$$a(\varpi_{0})\leq \lim_{a_{n}(\varpi_{0})\to a{\left(\varpi_{0}\right)}^{+}}W(a_{n}(\varpi_{0}))\leq W(a(\varpi_{0}))<a(\varpi_{0}),$$

a contradiction. Thus we get,

(32) $$\lim_{n\to \infty }a_{n}(\varpi )=0,$$

for all ϖ>0.

Similarly

(33) $$\lim_{n\to \infty }b_{n}(\varpi )=0,$$

for all ϖ>0.

Now, we prove that $\left\{(z_{n},w_{n})\right\}$ is a Cauchy bisequence. Suppose that $\left\{(z_{n},w_{n})\right\}$ is not Cauchy bisequence, then for some ϵ>0 there are two subsequences n(x) and m(x) of $Z^{+}$ with n(x)>m(x)>x.

(34) $$B(z_{n(x)},w_{m(x)},\varpi )\geq \epsilon ,$$

for all ϖ>0.

n(x) is selected in such a way that it is the smallest integer satisfying equation (34), it means

(35) $$B(z_{n(x)-1},w_{m(x)},\varpi )\leq \epsilon ,$$

for all ϖ>0.

So,

$$\epsilon \leq B(z_{n(x)},w_{m(x)},\varpi )\leq B(z_{n(x)},w_{n(x)-1},\varpi )\;o\;B(z_{n(x)-1},w_{n(x)-1},\varpi )\;o\;B(z_{n(x)-1},w_{m(x)},\varpi ).$$

Taking limit x→∞ and using equations (32), (33), (35) and ao0=a above becomes

(36) $$\lim_{x\to \infty }B(z_{n(x)},w_{m(x)},\varpi )=\epsilon .$$

By the Definition of gpbms

$$\epsilon \leq B(z_{n(x)},w_{m(x)},\varpi )\leq B(z_{n(x)},w_{n(x)},\frac{\varpi }{3}\;o\;B(z_{m(x)+1},w_{n(x)},\frac{\varpi }{3})\;o\;B(z_{m(x)},w_{m(x)},\frac{\varpi }{3}),$$

and

$$B(z_{m(x)+1},w_{n(x)},\frac{\varpi }{3})\leq B(z_{m(x)+1},w_{m(x)},\frac{\varpi }{9}\;o\;B(z_{n(x)},w_{m(x)},\frac{\varpi }{9})\;o\;B(z_{n(x)},w_{n(x)},\frac{\varpi }{9}).$$

Taking x→∞ and using equations (32), (33), (36), the above becomes

(37) $$\lim_{x\to \infty }B(z_{m(x)+1},w_{n(x)},\frac{\varpi }{3})=\epsilon ,$$

for all $\frac{\varpi }{3}>0\Rightarrow \varpi >0$.

Now,

(38) $$B(z_{m(x)+1},w_{n(x)},\varpi )=B(z_{n(x)+1},w_{m(x)},\varpi )\leq W(M(z_{n(x)},w_{m(x)},\varpi )),$$

where

$$M(z_{n(x)},w_{m(x)},\varpi )=\max\{B(z_{n(x)},w_{m(x)},\varpi ),B(z_{n(x)},Fz_{n(x)},\varpi ),B(Fw_{m(x)},w_{m(x)},\varpi ),\frac{B(z_{n(x)},Fz_{n(x)},\varpi )+B(Fw_{m(x)},w_{m(x)},\varpi )}{2},\frac{B(z_{n(x)},Fz_{n(x)},\varpi )\cdot B(Fw_{m(x)},w_{m(x)},\varpi )}{B(z_{n(x)},w_{m(x)},\varpi )},\frac{B(z_{n(x)},Fz_{n(x)},\varpi )(1+B(Fw_{m(x)},w_{m(x)},\varpi ))}{1+B(z_{n(x)},w_{m(x)},\varpi )}\},=\max\{B(z_{n(x)},w_{m(x)},\varpi ),B(z_{n(x)},w_{n(x)},\varpi ),B(z_{m(x)+1},w_{m(x)},\varpi ),\frac{B(z_{n(x)},w_{n(x)},\varpi )+B(z_{m(x)+1},w_{m(x)},\varpi )}{2},\frac{B(z_{n(x)},w_{n(x)},\varpi )\cdot B(z_{m(x)+1},w_{m(x)},\varpi )}{B(z_{n(x)},w_{m(x)},\varpi )},\frac{B(z_{n(x)},w_{n(x)},\varpi )(1+B(z_{m(x)+1},w_{m(x)},\varpi ))}{1+B(z_{n(x)},w_{m(x)},\varpi )}\}.$$

Using this in (38) and equation (37), taking x→∞, we get

ϵ≤W(max{ϵ,0,0,0,0,0})=ϕ(ϵ)<ϵ,

a contradiction.

So, $\left\{(z_{n},w_{n})\right\}$ is a Cauchy bisequence. Since the space is complete, hence by Proposition 2.1, $\left\{(z_{n},w_{n})\right\}$ is biconvergent that is there exists u∈G∩H such that $z_{n}\to u$ and $w_{n}\to u$ as n→∞.

Now, by continuity of F, we have $\lim_{n\to \infty }z_{n}=u$ implies

(39) $$\lim_{n\to \infty }Fz_{n}=\mathrm{Fu}=\lim_{n\to \infty }w_{n}=u.$$

So, equation (39) implies that Fu=u.

Uniqueness: If possible suppose that *u* and *v* are distinct fixed points.

(40) B(u,v,ϖ)=B(Fu,Fv,ϖ)≤W(M(u,v,ϖ)),

where

$$M(u,v,\varpi )=\max\{B(u,v,\varpi ),B(u,Fu,\varpi ),B(Fv,v,\varpi ),\frac{B(u,Fu,\varpi )+B(Fv,v,\varpi )}{2},\frac{B(u,Fu,\varpi )\cdot B(Fv,v,\varpi )}{B(u,v,\varpi )},\frac{B(u,Fu,\varpi )(1+B(Fv,v,\varpi ))}{1+B(u,v,\varpi )}\},=\max\{B(u,v,\varpi ),0,0,0,0\}.$$

Putting in (40), we get

B(u,v,ϖ)≤W(B(u,v,ϖ))<B(u,v,ϖ),

a contradiction.

This implies that u=v. □

Example 2.7 Let G=[−119,0], H=[0,119] and define B:G×H×(0,∞)→[0,∞) as $B(z,w,\varpi )=\frac{|z-w{|}^{p}}{\varpi }$ for all ϖ>0 and $p\in (0,\frac{1}{2})$.

Define $a\;o\;b={\left(a^{2}+b^{2}\right)}^{\frac{1}{2}}$.

So, (G,H,B,o) is a complete gpbms.

Taking $Fz=\frac{13z}{97}$ and $W(\delta )={\left(\frac{14}{97}\right)}^{p}\delta$.

As, F([−119,0])=[−15.9,0]⊆[−119,0] and F([0,119])=[0,15.9]⊆[0,119].

So, F is a covariant map.

$$B(Fz,Fw,\varpi )=B(\frac{13z}{97},\frac{13w}{97},\varpi )={\left(\frac{13}{97}\right)}^{p}\frac{|z-w{|}^{p}}{\varpi },\leq {\left(\frac{14}{97}\right)}^{p}\frac{|z-w{|}^{p}}{\varpi },=W(B(z,w,\varpi )).$$

So, equation (1) is satisfied.

Since, W∈Σ.

So, all the conditions of Theorem 2.3 are satisfied. Hence, F has a unique fixed point.

Clearly, 0 is unique fixed point of F.

Example 2.8 Let G=[−111,0], H=[0,111] and define B:G×H×(0,∞)→[0,∞) as $B(z,w,\varpi )=\frac{|z-w{|}^{p}}{\varpi }$ for all ϖ>0 and $p\in (0,\frac{1}{2})$.

Define $a\;o\;b={\left(a^{2}+b^{2}\right)}^{\frac{1}{2}}$.

So, (G,H,B,o) is a complete gpbms.

Taking $Fz=-\frac{15z}{83}$ and $W(\delta )={\left(\frac{17}{83}\right)}^{p}\delta$.

As, F([−111,0])=[0,20.06]⊆[0,111] and F([0,111])=[−20.06,0]⊆[−111,0].

So, F is a contravariant map.

$$B(Fw,Fz,\varpi )=B(\frac{15w}{83},\frac{15z}{83},\varpi )={\left(\frac{15}{83}\right)}^{p}\frac{|z-w{|}^{p}}{\varpi },\leq {\left(\frac{17}{83}\right)}^{p}\frac{|z-w{|}^{p}}{\varpi },=W(B(z,w,\varpi )).$$

So, equation (6) is satisfied.

Since, W∈Σ.

So, all the conditions of Theorem 2.4 are satisfied. Hence, F has a unique fixed point.

Clearly, 0 is unique fixed point of F.

Example 2.9 Let G={0,1,2}, H={2,3} and define B:G×H×(0,∞)→[0,∞) as $B(z,w,\varpi )=\frac{|z-w{|}^{p}}{\varpi }$ for all ϖ>0 and $p\in (0,\frac{1}{2})$.

Define aob=a+b.

So, (G,H,B,o) is a complete gpbms.

Let $W(\delta )=\frac{1}{2^{p}}\delta$ and F({0,1,2})=2 and F(3)=1.

So, F is a contravariant map.

Now, to evaluate the right hand side of equation (13), we need the values of M(z,w,ϖ). Thus value of each term of M(z,w,ϖ) for the above settings is represented by the above Table 1. From the Table 1 equation (13) holds clearly for all (z,w)∈(G,H).

**Table 1:** Values of M(z,w,ϖ).

(z,w)	B(Fw,Fz,ϖ)	B(z,w,ϖ)	B(z,Fz,ϖ)	B(Fw,w,ϖ)	M(z,w,ϖ)
(0,2)	0	$\frac{2^{p}}{\varpi }$	$\frac{2^{p}}{\varpi }$	0	$\frac{2^{p}}{\varpi }$
(0,3)	$\frac{1}{\varpi }$	$\frac{3^{p}}{\varpi }$	$\frac{2^{p}}{\varpi }$	$\frac{2^{p}}{\varpi }$	$\frac{3^{p}}{\varpi }$
(1,2)	0	$\frac{1}{\varpi }$	$\frac{1}{\varpi }$	0	$\frac{1}{\varpi }$
(1,3)	$\frac{1}{\varpi }$	$\frac{2^{p}}{\varpi }$	$\frac{1}{\varpi }$	$\frac{2^{p}}{\varpi }$	$\frac{2^{p}}{\varpi }$
(2,2)	0	0	0	0	0
(2,3)	$\frac{1}{\varpi }$	$\frac{1}{\varpi }$	0	$\frac{2^{p}}{\varpi }$	$\frac{2^{p}}{\varpi }$

Since, F is continuous. So, all the conditions of Theorem 2.5 holds. Hence, F has a unique fixed point.

Clearly, 2 is the unique fixed point of F.

Example 2.10 Let G={0,1,2}, H={2,3} and define B:G×H×(0,∞)→[0,∞) as $B(z,w,\varpi )=\frac{|z-w{|}^{p}}{\varpi }$ for all ϖ>0 and $p\in (0,\frac{1}{2})$.

Define aob=a+b.

So, (G,H,B,o) is a complete gpbms.

Let $W(\delta )=\frac{1}{2^{p}}\delta$ and F({0,1,2})=2 and F(3)=1.

So, F is a contravariant map.

To verify the equation (27), we need to evaluate the value of M(z,w,ϖ). So, value of each term of M(z,w,ϖ) is represented by Table 2, Table 3.

**Table 2:** Values of each term of M(z,w,ϖ).

(z,w)	B(Fw,Fz,ϖ)	B(z,w,ϖ)	B(z,Fz,ϖ)	B(Fw,w,ϖ)	$\frac{B(z,Fz,\varpi )+B(Fw,w,\varpi )}{2}$
(0,2)	0	$\frac{2^{p}}{\varpi }$	$\frac{2^{p}}{\varpi }$	0	$\frac{2^{p-1}}{\varpi }$
(0,3)	$\frac{1}{\varpi }$	$\frac{3^{p}}{\varpi }$	$\frac{2^{p}}{\varpi }$	$\frac{2^{p}}{\varpi }$	$\frac{2^{p}}{\varpi }$
(1,2)	0	$\frac{1}{\varpi }$	$\frac{1}{\varpi }$	0	$\frac{1}{2\varpi }$
(1,3)	$\frac{1}{\varpi }$	$\frac{2^{p}}{\varpi }$	$\frac{1}{\varpi }$	$\frac{2^{p}}{\varpi }$	$\frac{1+2^{p}}{2\varpi }$
(2,2)	0	0	0	0	0
(2,3)	$\frac{1}{\varpi }$	$\frac{1}{\varpi }$	0	$\frac{2^{p}}{\varpi }$	$\frac{2^{p-1}}{\varpi }$

**Table 3:** Continued values of each term of M(z,w,ϖ).

(z,w)	$\frac{B(z,Fz,\varpi )\cdot B(Fw,w,\varpi )}{B(z,w,\varpi )}$	$\frac{B(z,Fz,\varpi )(1+B(Fw,w,\varpi )}{1+B(z,w,\varpi )}$	M(z,w,ϖ)
(0,2)	0	$\frac{\frac{2^{p}}{\varpi }}{1+\frac{2^{p}}{\varpi }}$	$\frac{2^{p}}{\varpi }$
(0,3)	$\frac{2^{2p}}{3^{p}\varpi }$	$\frac{\frac{2^{p}}{\varpi }(1+\frac{2^{p}}{\varpi })}{1+\frac{3^{p}}{\varpi }}$	$\frac{3^{p}}{\varpi }$
(1,2)	0	$\frac{\frac{1}{\varpi }}{1+\frac{1}{\varpi }}$	$\frac{1}{\varpi }$
(1,3)	$\frac{1}{\varpi }$	$\frac{1}{\varpi }$	$\frac{2^{p}}{\varpi }$
(2,2)	0	0	0
(2,3)	0	0	$\frac{2^{p}}{\varpi }$

From the Table 2, Table 3, equation (27) holds clearly for all (z,w)∈(G,H).

Since, F is continuous. So, all the conditions of Theorem 2.6 hold. Hence, F has a unique fixed point.

Clearly, 2 is the unique fixed point of F.

Example 2.11 Let $G=\{(x,0)\in R^{2}:x\in [0,\infty )\}$, $H=\{(0,y)\in R^{2}:y\in [0,\infty )\}$ and define B:G×H×(0,∞)→[0,∞) as $B(z,w,\varpi )=\frac{|z_{1}-w_{2}{|}^{p}}{\varpi }$ for all ϖ>0 and $p\in (0,\frac{1}{2})$, where $z=(z_{1},z_{2})\in G$ and $w=(w_{1},w_{2})\in H$.

Define $a\;o\;b={\left(a^{2}+b^{2}\right)}^{\frac{1}{2}}$.

So, (G,H,B,o) is a complete gpbms.

Taking

$$F(z,w=\left\{ \begin{array}{cc} \left(\frac{3}{7}z,0\right) & \text{if }(z,w)\in G\\ \left(0,\frac{3}{7}w\right) & \text{if }(z,w)\in H \end{array}\right.$$

$W(\delta )={\left(\frac{6}{7}\right)}^{p}\delta$.

As, F(G)⊆G and F(H)⊆H.

So, F is a covariant map.

$$B(Fz,Fw,\varpi )=\frac{|\frac{3}{7}z_{1}-\frac{3}{7}w_{2}{|}^{p}}{\varpi }={\left(\frac{3}{7}\right)}^{p}.\frac{|z_{1}-w_{2}{|}^{p}}{\varpi }\leq {\left(\frac{6}{7}\right)}^{p}.\frac{|z_{1}-w_{2}{|}^{p}}{\varpi }=W(B(z,w,\varpi )).$$

So, equation (1) is satisfied.

Since, W∈Σ.

So, all the conditions of Theorem 2.3 are satisfied. Hence, F has a unique fixed point.

Clearly, (0,0) is unique fixed point of F.

## 3 Consequences

In this section we shall discuss about some consequences of our main results.

Corollary 1 *Let* (G,H,B,o) *be a complete gpbms and* F:(G,H)⇉(G,H) *is a covariant map such that*

(41) B(Fz,Fw,ϖ)≤k.B(z,w,ϖ),

*for all* ϖ>0 *,* (z,w)∈G×H *and* k∈[0,1) *for all* ϖ>0 *.*

Proof By inserting W(ϖ)=k.ϖ in equation (1), one can obtain the equation (41). So, proof can be easily deduced from Theorem 2.3. □

Corollary 2 *Let* (G,H,B,o) *be a complete gpbms and* F:(G,H)⇄(G,H) *is a covariant map such that*

(42) B(Fw,Fz,ϖ)≤k.B(z,w,ϖ),

*for all* ϖ>0 *,* (z,w)∈G×H *and* k∈[0,1) *for all* ϖ>0 *.*

Proof By inserting W(ϖ)=k.ϖ in equation (6), one can obtain the equation (42). So, proof is the direct consequence of Theorem 2.4. □

## 4 Applications

In this section, we solve an integral as well as fractional differential equation.

Theorem 4.1 *Assume the integral equation*

(43) $$Z(\zeta )=w(\zeta )+\lambda_{1}\int_{D_{1}\cup D_{2}}\Lambda (\zeta ,\xi ,Z(\xi ))d\xi +\lambda_{2}\int_{D_{1}\cup D_{2}}\Lambda (\zeta ,\xi ,Z(\xi ))d\xi +...+\lambda_{n}\int_{D_{1}\cup D_{2}}\Lambda (\zeta ,\xi ,Z(\xi ))d\xi ,\zeta \in D_{1}\cup D_{2},$$

*where*$D_{1}\cup D_{2}$*is a Lebesgue measurable set (*$D_{1}\cap D_{2}\neq \phi$*) and*$\lambda_{i}$*'s are different constants.*

*Suppose*

(1) $\Lambda :D_{1}^{2}\cup D_{2}^{2}\times [0,\infty )\to [0,\infty )$*and*$w(\zeta )\in L^{\infty }(D_{1})\cup L^{\infty }(D_{2})$*,*
(2) *Suppose*$\delta :D_{1}^{2}\cup D_{2}^{2}\to [0,\infty )$*is a continuous map such that*

|Λ(ζ,ξ,Z(ξ)−Λ(ζ,ξ,E(ξ)|≤δ(ζ,ξ)|Z(ξ)−E(ξ)|

*for all*i∈{1,2,...n}*, where*τ≥0*and*$\zeta ,\xi \in D_{1}^{2}\cup D_{2}^{2}$*, and (C1).*$(||\int_{D_{1}\cup D_{2}}\delta (\zeta ,\xi )|{|}_{\infty })d\xi \leq \frac{k}{\lambda }$*that is*$\sup_{D_{1}\cup D_{2}}\int_{D_{1}\cup D_{2}}|\delta (\zeta ,\xi )|d\xi \leq \frac{k}{\lambda .n}$*where*k∈[0,1)*and*$\lambda =\max\{\lambda_{1},\lambda_{2},...\lambda_{n}\}$*.*

*Then the integral equation*(43)*has a unique solution in*$L^{\infty }(D_{1})\cup L^{\infty }(D_{2})$*.*

Proof Let $G=L^{\infty }(D_{1})$ and $H=L^{\infty }(D_{2})$ are two normed linear spaces, where $D_{1},D_{2}$ are measurable sets with $m(D_{1}\cup D_{2})<\infty$. Define B:G×H×(0,∞)→[0,∞) as $B(\zeta ,\xi ,\varpi )=\frac{||\zeta -\xi |{|}_{\infty }}{\varpi }$ for ϖ>0 and ζoξ=ζ+ξ

Clearly, (G,H,B,o) is a complete gpbms.

Define a mapping τ:G∪H→G∪H as $\tau (Z(\zeta ))=w(\zeta )+\int_{D_{1}\cup D_{2}}\Lambda (\zeta ,\xi ,Z(\xi ))d\xi ,\zeta \in D_{1}\cup D_{2}$.

Since, $w(\zeta )\in L^{\infty }(D_{1})$ if $\zeta \in D_{1}$ and $w(\zeta )\in L^{\infty }(D_{2})$ if $\zeta \in D_{2}$.

So, *τ* is a covariant map.

Now,

$$B(\tau Z(\zeta ),\tau E(\zeta ))=\frac{||\tau Z(\zeta )-\tau E(\zeta )|{|}_{\infty }}{\varpi }=\frac{||w(\zeta )+\sum_{i=1}^{n}\lambda_{i}\int_{D_{1}\cup D_{2}}\Lambda (\zeta ,\xi ,Z(\xi ))d\xi -w(\zeta )-\sum_{i=1}^{n}\lambda_{i}\int_{D_{1}\cup D_{2}}\Lambda (\zeta ,\xi ,E(\xi ))d\xi |{|}_{\infty }}{\varpi }=\int_{D_{1}\cup D_{2}}\frac{\sum_{i=1}^{n}\lambda_{i}|(\Lambda (\zeta ,\xi ,Z(\xi ))-\Lambda (\zeta ,\xi ,E(\xi )))|d\xi }{\varpi }\leq \frac{1}{\varpi }\int_{D_{1}\cup D_{2}}\sum_{i=1}^{n}\lambda_{i}|\delta (\zeta ,\xi )|d\xi |Z(\zeta )-E(\zeta )|\leq \frac{(\lambda_{1}+\lambda_{2}+...+\lambda_{n})k}{\varpi (\lambda .n)}||Z(\zeta )-E(\zeta )|{|}_{\infty }=(\frac{\lambda_{1}}{\lambda }+\frac{\lambda_{2}}{\lambda }+...\frac{\lambda_{n}}{\lambda })\frac{k}{n.\varpi }||Z(\zeta )-E(\zeta )|{|}_{\infty }\leq kB(Z(\zeta ),E(\zeta ))$$

Hypothesis of Corollary 1 hold.

Hence H has a unique fixed point.

So, integral equation (43) has a unique solution. □

For a function k∈C[0,1], the Riemann-Liouville fractional derivative of order γ>0 is given by

$$\frac{1}{\Gamma (s-\gamma )}\frac{d^{s}}{d\sigma^{s}}\int_{0}^{\sigma }\frac{k(k)d\pi }{{\left(\sigma -\pi \right)}^{\gamma -s+1}}=D^{\gamma }k(\sigma ),$$

provided that right hand side is pointwise defined on [0,1], where [γ] is the integral part of number *γ*, Γ is the Euler gamma function. Consider the following fractional differential equation

(44) Dμπk(σ)+f(σ,k(σ))=0,0≤σ≤0,2≤μ>1;k(0)=k(1)=0,

where F is a continuous function from [0,1]×R to R and Dμπ represents the Caputo fractional derivative of order *μ* and it is defined by

Dμπ=1Γ(s−μ)∫0kks(π)dπ(σ−π)μ−s+1

Theorem 4.2 *Consider fractional differential equation* (44) *. Suppose that if*

(1) *There exists* σ∈[0,1] *,* ρ∈(0,1) *and* $(k,k^{\prime })\in G\times H$ *such that*
  $$|f(\sigma ,k)-f(\sigma ,k^{\prime }){|}^{p}\leq \rho^{\frac{1}{p}}{\left(|k(\sigma )-k(\sigma^{\prime })|\right)}^{p},$$
  *where* G=(C[0,1],[0,∞)) *and* H=(C[0,1],(−∞,0]) *and* f *is non decreasing in* [0,1] *.*
(2) $\sup_{\sigma \in [0,1]}\int_{0}^{1}|G(\sigma ,e){|}^{p}de\leq 1$ *.*

*Then, equation* (44) *has a unique solution in* G∪H *.*

Proof Let G=(C[0,1],[0,∞)) that is set of all continuous function on [0,1] taking values in [0,∞) and H=(C[0,1],(−∞,0]) that is set of all continuous function on [0,1] taking values in (−∞,0]).

Define $B(z,w,\varpi )=\sup_{\sigma \in [0,1]}\frac{{\left(|z(\sigma )-w(\sigma )|\right)}^{p}}{\varpi }$ where 0<p<1 and ϖ>0.

Taking $a\;o\;b={\left(a^{2}+b^{2}\right)}^{\frac{1}{2}}$.

Then, (G,H,B,o) is a complete gpmbms.

Equation (44) can be rewritten as $k(\sigma )=\int_{0}^{1}G(\sigma ,e)f(B,k(e))de$, where

$$G(\sigma ,e)=\left\{ \begin{array}{cc} \frac{{\left[\sigma (1-e)\right]}^{\mu -1}-{\left(\sigma -e\right)}^{\mu -1}}{\Gamma (\mu )} & \text{if }0\leq e\leq \sigma \leq 1,\\ \frac{{\left(\sigma (1-e)\right)}^{\mu -1}}{\Gamma (\mu )} & \text{if }0\leq \sigma \leq e\leq 1. \end{array}\right.$$

Define the mapping F:G∪H→G∪H as

$$Fk(\sigma )=\int_{0}^{1}G(\sigma ,e)f(B,k(e))de.$$

Since f is continuous on [0,1]×R and non decreasing on [0,1], so F is a covariant mapping.

It is easy to verify that if w is the fixed point of F then it is the solution of equation (44). Now

$${\left(|Fk(\sigma )-Fk^{\prime }(\sigma )|\right)}^{p}=|\int_{0}^{1}G(\sigma ,e)f(B,k(e))de-\int_{0}^{1}G(\sigma ,e)f(B,k^{\prime }(e))de{|}^{p}=\int_{0}^{1}{\left(|G(\sigma ,e)|\right)}^{p}de.\int_{0}^{1}{\left(|f(B,k(e))-f(B,k^{\prime }(e))|\right)}^{p}de\leq \rho {\left(|k(\sigma )-k^{\prime }(\sigma )|\right)}^{p}\;\frac{{\left(|Fk(\sigma )-Fk^{\prime }(\sigma )|\right)}^{p}}{\varpi }\leq \rho \frac{{\left(|k(\sigma )-k^{\prime }(\sigma )|\right)}^{p}}{\varpi }$$

Taking sup on both sides, we get

$$B(Fk(\sigma ),Fk^{\prime }(\sigma ),\varpi )\leq \rho (B(k(\sigma ),k^{\prime }(\sigma ),\varpi )),$$

for all ϖ>0. So, all the conditions of Corollary 1 are satisfied. Hence, F has a unique fixed point. Thus, equation (44) has a unique solution in G∪H. □

## 5 Conclusion

In this paper, we have introduced the new notion of generalized parametric bipolar metric space and proved some fixed point results for the Boyd-Wong type contractions in the above mentioned space. Then, we provide some non trivial examples to illustrate the main results. We have solved an integral as well as fractional differential equations with the help of our proved results. It is an open problem for the researchers to check whether Theorem 2.5, Theorem 2.6 can also be proved for covariant maps. They can also extend our results in other metric spaces like $C^{⁎}$-algebra valued bipolar metric space, fuzzy bipolar metric space, soft bipolar metric space etc.

## CRediT authorship contribution statement

**Manoj Kumar:** Writing – review & editing, Writing – original draft, Validation, Investigation, Formal analysis, Conceptualization. **Ozgur Ege:** Writing – review & editing, Writing – original draft, Validation, Supervision, Methodology, Investigation, Formal analysis, Conceptualization. **Vinit Mor:** Writing – original draft, Validation, Investigation, Formal analysis. **Pankaj Kumar:** Writing – original draft, Validation, Investigation, Formal analysis. **Manuel De la Sen:** Writing – review & editing, Writing – original draft, Validation, Supervision, Project administration, Funding acquisition.

## Declaration of Competing Interest

The authors declare the following financial interests/personal relationships which may be considered as potential competing interests: Manuel De la Sen reports financial support was provided by 10.13039/501100003086Basque Government.

## Data Availability

No data was used for the research described in the article.
